# Prediction Models for the Mechanical Properties of Self-Compacting Concrete with Recycled Rubber and Silica Fume

**DOI:** 10.3390/ma13081821

**Published:** 2020-04-12

**Authors:** Robert Bušić, Mirta Benšić, Ivana Miličević, Kristina Strukar

**Affiliations:** 1 Faculty of Civil Engineering and Architecture Osijek, Josip Juraj Strossmayer University of Osijek, Vladimira Preloga 3, HR-31000 Osijek, Croatia; ivana.milicevic@gfos.hr (I.M.); kstrukar@gfos.hr (K.S.); 2 Department of Mathematics, Josip Juraj Strossmayer University of Osijek, Gajev trg 6, HR-31000 Osijek, Croatia; mirta@mathos.hr

**Keywords:** self-compacting rubberized concrete (SCRC), waste tire rubber, silica fume, fresh and hardened SCRC properties, multivariate regression models

## Abstract

The paper aims to investigate the influence of waste tire rubber and silica fume on the fresh and hardened properties of self-compacting concrete (SCC) and to design multivariate regression models for the prediction of the mechanical properties of self-compacting rubberized concrete (SCRC). For this purpose, 21 concrete mixtures were designed. Crumb rubber derived from end-of-life tires (grain size 0.5–3.5 mm) was replaced fine aggregate by 0%, 5%, 10%, 15%, 20%, 25%, and 30% of total aggregate volume. Silica fume was replaced cement by 0%, 5%, and 10% of the total cement mass. The optimal replacement level of both materials was investigated in relation to the values of the fresh properties and mechanical properties of self-compacting concrete. Tests on fresh and hardened self-compacting concrete were performed according to the relevant European standards. Furthermore, models for predicting the values of the compressive strength, modulus of elasticity, and flexural strength of SCRC were designed and verified with the experimental results of 12 other studies. According to the obtained results, mixtures with up to 15% of recycled rubber and 5% of silica fume, with 28 days compressive strength above 30 MPa, were found to be optimal mixtures for the potential future investigation of reinforced self-compacting rubberized concrete structural elements.

## 1. Introduction

After almost two decades, the idea to combine concrete with waste recycled tire rubber as an aggregate replacement is still the subject of a large number of experimental investigations. To date, a considerable number of authors investigated traditional concrete with recycled rubber derived from end-of-life tires (ELTs), and it is known that this type of environmentally friendly concrete has been used in non-structural concrete elements such as traffic noise barriers, sidewalks, and sport fields [1,2,3,4] mainly because the implementation of the waste tire rubber in concrete reduces the value of the compressive strength and modulus of elasticity of concrete [5,6,7,8,9,10,11,12,13]. On the other hand, a smaller number of authors investigated the self-compacting rubberized concrete (SCRC) in terms of concrete used for structural elements [8,14,15,16]. The advantages of using recycled rubber as a substitute material for natural fine and/or coarse aggregate in concrete such as increased ductility, reduced total concrete mass, improved dynamic properties [17,18,19,20,21,22], increased freeze–thaw resistance [23,24,25,26,27], increased cracking resistance [16,28], increased fracture energy [22,29,30], and concrete resistance to tensile stresses [30] provide sufficient reasons to continue experimental research and laboratory work on reinforced concrete elements and system (columns, beams, frames) with a certain percentage of recycled rubber powder and granules as a replacement material for fine or coarse aggregate. From the previous experimental work [6,9,31,32] and literature reviews [33,34,35], it can be concluded that when replacing natural fine aggregate with rubber granules, the negative impact on mechanical characteristics of the material is still lesser compared to the negative impact of replacing natural coarse or natural fine and coarse aggregate with rubber aggregate.

The mechanical properties of SCRC can vary significantly depending on mixture design, material properties, supplementary cementitious materials (SCMs) used in experiment, type and size of recycled rubber, etc. The use of crumb rubber (CR) and SCMs in structural self-compacting concrete (SCC) is a promising method to decrease the negative impacts of waste on the environment and to reduce cement consumption. Before structural application and full-scale experiments in the laboratory, the SCC mixture design needs to be determined, due to the desired concrete strength. In addition to the requirements for the mechanical resistance and stability of the structure, the replacement level of CR and SCMs should also be selected respecting economic criteria, since the replacement of the natural fine aggregate with crumb rubber negatively effects on the designed fresh and hardened state properties of SCC. Hence, the use of mineral and chemical additives, such as different types of SCMs and superplasticizers, are obligatory to preserve concrete design properties. SCMs are mostly used to enhance the mechanical and durability properties of SCC. Recently, there are many studies on the effect of silica fume (SLF) and other SCMs on the fresh and hardened properties of highly flowable cementitious composites. Some of these studies investigated the mutual effects of SLF and other SCMs, such as fly ash, metakaolin (MK), and ground granulated blast-furnace slag (GGBFS) on the mechanical and durability properties [36,37,38,39,40] of highly flowable cementitious composites, while other studies investigated the individual effect of SLF on SCC properties [41,42,43]. Considering SCRC, in several experiments where the mutual effects of SLF and other SCMs were investigated, an improvement in mechanical properties was reported [6,44]. Furthermore, AbdelAleem et al. [5] investigated how a 10% replacement level of cement with SLF influences SCRC mechanical properties. However, there are no studies on the combination effect of different replacement levels of both CR and SLF, without any other SCMs, on the fresh and hardened properties of SCC.

There are two main research objectives of the present study. First, (i) to examine the coupling effect of different replacement levels of both SLF and CR on the mechanical properties of SCC. For this purpose, 21 SCC mixtures were made, with different replacement levels of natural fine aggregate (0%, 5%, 10%, 15%, 20%, 25%, 30% of total aggregate volume) with CR and cement (0%, 5%, and 10% by mass) with SLF. Second, (ii) to develop prediction models for the mechanical properties of SCC with CR and SLF, i.e., compressive strength, modulus of elasticity, and flexural strength. In Section 2 and Section 3, the materials, methods, and test results are given. Furthermore, appropriate models are presented and verified in Section 3 for each observed mechanical property by using two independent variables: the amount of CR and SLF.

## 2. Materials and Methods

### 2.1. Materials Used for Experiment

SCC mixtures were designed in accordance with the parameters given by the European Guidelines for self-compacting concrete [45]. Ordinary Portland cement CEM I 42.5R from the local cement factory Našice, Croatia, with a specific gravity of 3.17 g/cm^3^ and Blaine fineness of 4378 cm^2^/g, which complies with the requirement of European Standards EN 197-1:2002 [46], was used in this study. The chemical properties of cement used in experiment are given in Table 1. The chemical and physical properties are in accordance with the limit values given in EN 197-1:2002 [46]. Silica fume obtained from Pocking, Germany with the specific gravity of 2.2 g/cm^3^ and Blaine fineness of 20.5 m^2^/g, which is in accordance with the European Standards EN 13263-1:2009 [47], was used in concrete mixtures as a mineral additive and supplementary cementitious material. The properties of the silica fume used in the experimental study are given in Table 2. Tap water from the public water supply system, which is in accordance with EN 1008:2002 [48], was used as the mixing water of mixture. In order to achieve the desirable properties of self-compacting concrete in fresh state, i.e., flowability, viscosity, and passing ability classes, superplasticizer (SP) Energy FM500 and viscosity-modifying admixture (VMA) Premadd Stabilisator X were used in the production of SCC as chemical admixtures. The dosage of SP and VMA given by producers are between 1–2% and 0.2–0.3% of the total binder amount by weight, respectively. Both chemical admixtures are in compliance with EN 934-1:2008 [49] and EN 934-2:2012 [50]. If it was necessary, the amount of superplasticizer and viscosity-modifying admixture was adjusted during the mixing procedure, primarily to achieve acceptable slump flow values. The main properties of the chemical admixtures are given in Table 3, which was provided by the manufacturer.

Dolomite powder, size < 0.063 mm, from a local quarry, with a specific gravity of 2.97 g/cm^3^ and Blaine fineness of 5206 cm^2^/g was used as a filler. The density of cement, silica fume, and dolomite powder was tested according to ASTM C188-16:2011 [51].

**Figure 1:**
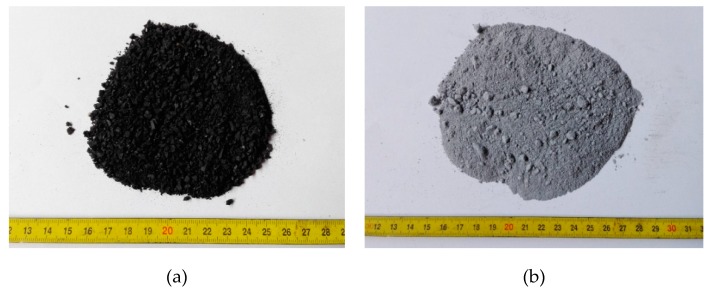
Test materials: (**a**) crumb rubber, (**b**) silica fume.

**Figure 2:**
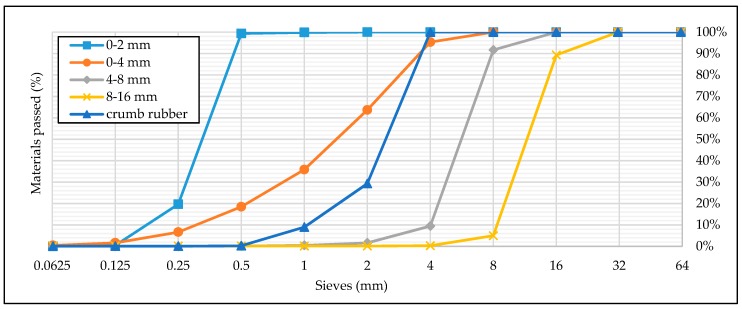
Sieve analysis of fine aggregate, coarse aggregate, and crumb rubber.

Natural sand and crushed stone with nominal sizes of 0–2, 0–4, 4–8, and 8–16 mm with specific gravities of 2.58, 2.79, 2.88, and 2.88 g/cm^3^ were used as a fine (FA) and coarse aggregate (CA). Rubber aggregate, size 0.5–3.5 mm, obtained by the mechanical grinding of local waste tires, was used as a fine aggregate replacement (0–4 mm). The specific gravity of rubber aggregate, i.e., crumb rubber (CR), was 1.05 g/cm^3^. The appearance of silica fume and crumb rubber is shown in Figure 1. The size grading of the fine aggregate, coarse aggregate, and crumb rubber, obtained through the sieve analysis according to EN 933-1:2012 [52], is shown in Figure 2.

### 2.2. Self-Compacting Concrete Mixtures Design

A total of 21 self-compacting concrete mixtures were tested in fresh and hardened state, regarding two variables: crumb rubber and silica fume. Reference mixture with 0% of crumb rubber and 0% of silica fume was marked as SCC-0CR-0SLF. Three replacement levels of the cement with SLF (0%, 5%, and 10% by total cement mass) and seven replacement levels of the FA with CR (0%, 5%, 10%, 15%, 20%, 25%, and 30% by total aggregate volume) were investigated. A summary of the SCC mixtures is listed in Table 4. SCC mixtures were designed having a constant water to binder (w/b) ratio of 0.4 and a total binder content of 450 kg/m^3^. The water to powder ratio (w/p) was 1.07, 1.05, and 1.03 for SCC mixtures with 0%, 5%, and 10% silica fume, respectively, and these values were in accordance with the permitted values given by European Guidelines for SCC [45]. For the reference SCC mixture, the percentage share of particular aggregate fractions of 0–2, 0–4, 4–8 and 8–16 mm was 20%, 35%, 20%, and 25% of total amount of aggregate volume, respectively. The dosage of SP was kept constant for all mixtures, while VMA was used only in concrete mixtures without silica fume, where slight segregation was observed.

### 2.3. Specimen Preparation and Curing Conditions

**Figure 3:**
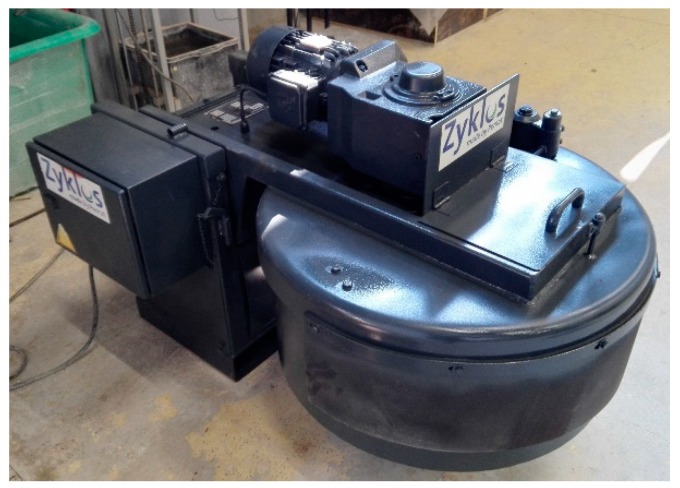
Pan mixer (50 L capacity).

**Figure 4:**
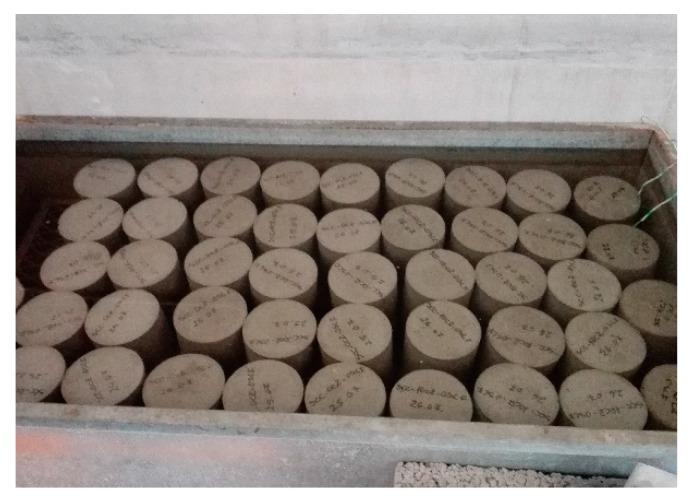
Pan mixer (50 L capacity).

Designed SCC mixtures were stirred with a pan mixer that had a maximal capacity of 50 L (Figure 3). At the beginning, natural fine and coarse aggregate were mixed homogeneously for 120 s, along with the water needed to have the natural aggregate saturated surface dry (SSD). The second step was to add silica fume, recycled crumb rubber, cement, filler, and about 75% of the total water volume, and the mixing was continued for another 2 min. The third and final step was to add chemical admixtures, superplasticizer, and a viscosity-modifying admixture, along with the remaining 25% of total water volume, and the SCC was mixed for an additional 4 min. Upon completion of the mixing process, fresh SCC properties were tested as described in Chapter 2.4. After testing the fresh SCC properties, concrete was cast into standardized molds, cylinders, and prisms, without any vibration or compaction, and protected with plastic foil for the next 24 h. All test specimens were demolded 24 h after the casting and placed in a water tank for 4 weeks (Figure 4).

The hardened state properties of SCC were determined after 28 days of water curing.

### 2.4. Test Methods

**Figure 5:**
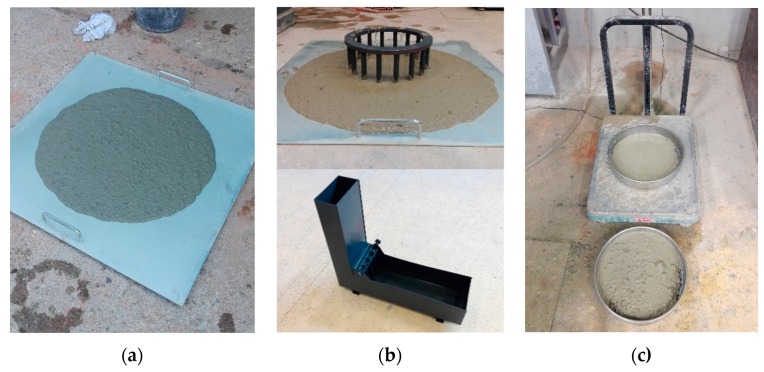
Test of fresh self-compacting concrete: (**a**) flowability and viscosity (slump flow test); (**b**) passing ability (J-ring and L-box); (**c**) segregation resistance (sieve segregation test).

Tests on fresh and hardened SCC were performed according to relevant European standards. The J-ring test, L-box test, slump flow test, and segregation resistance test were performed according to EN 12350-12:2010 [53], EN 12350-10:2010 [54], EN 12350-8:2010 [55], and EN 12350-11:2010 [56], respectively. Flowability and viscosity of SCC were measured and classified through the slump flow test (Figure 5a). Passing ability was measured and classified through two different methods, the L-box and J-ring tests (Figure 5b), while segregation resistance was measured and classified through the sieve segregation test (Figure 5c).

**Figure 6:**
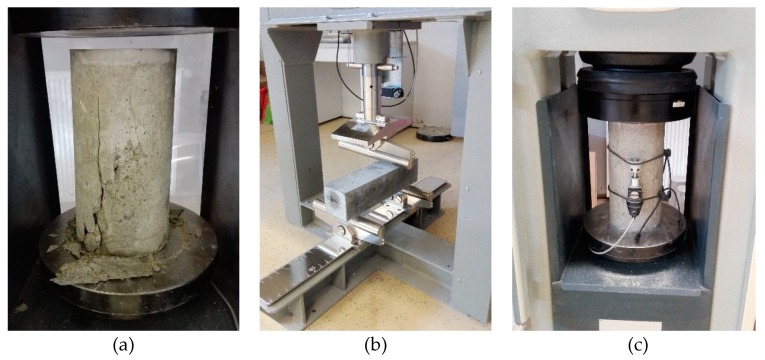
Test of hardened self-compacting concrete: (**a**) compressive strength; (**b**) flexural strength; (**c**) modulus of elasticity.

Compressive strength (f_ck,cyl_) was determined through testing cylinders (Figure 6a) with the dimensions of Ø150 × 300 mm using EN 12390-3:2009 [57]. Flexural strength (f_b_) was tested on 100 × 100 × 400 mm prisms (Figure 6b) according to EN 12390-5:2009 [58]. The modulus of elasticity (E) was determined through testing cylinder specimens (Figure 6c) with the dimensions of Ø150 × 300 mm using EN 12390-13:2013 [59].

According to percentage values of the two main variables, i.e., silica fume and crumb rubber, a total of 126 cylinder specimens and 63 prism specimens were prepared. Three specimens were prepared for the same set of study parameters. The obtained results of compressive strength (f_ck, cyl_), modulus of elasticity (E), and flexural strength (f_b_) were presented as the average of three samples. A summary of the tested properties of SCC in fresh and hardened states is given in Table 5.

## 3. Results

### 3.1. Fresh SCC Properties

**Figure 7:**
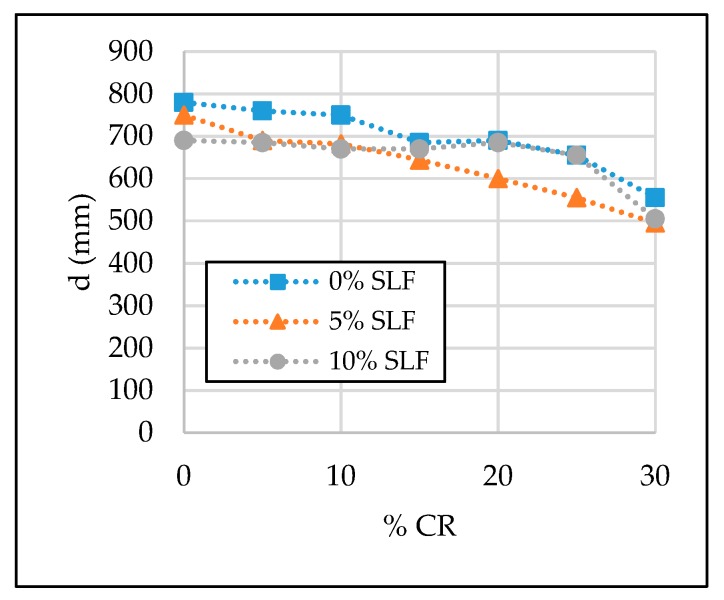
The effect of crumb rubber and silica fume on slump flow diameter.

**Figure 8:**
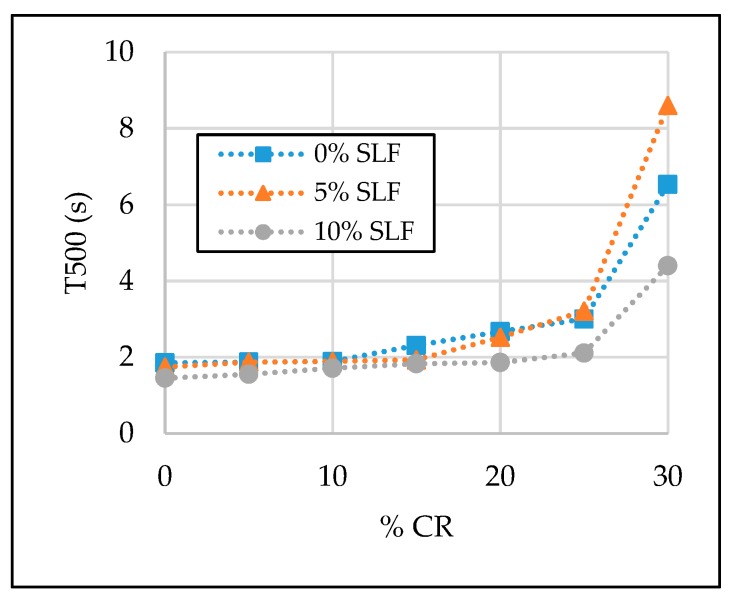
The effect of crumb rubber and silica fume on T500 time.

**Figure 9:**
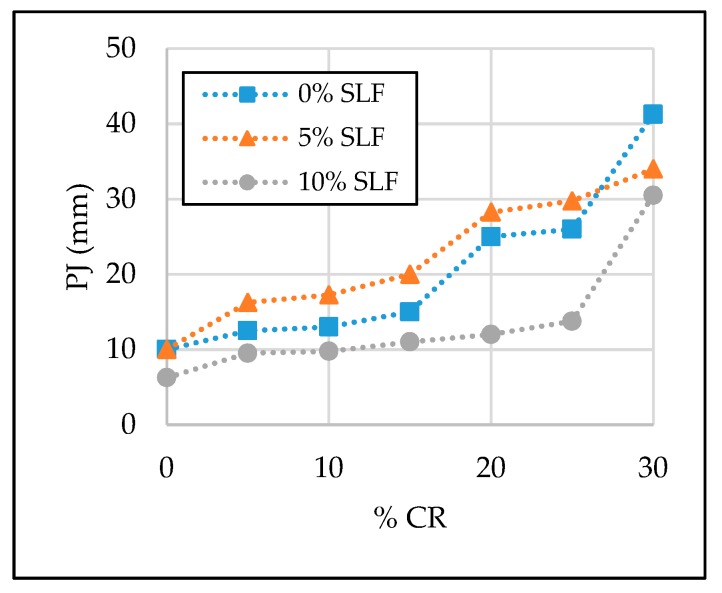
The effect of crumb rubber and silica fume on J-ring value (PJ).

**Figure 10:**
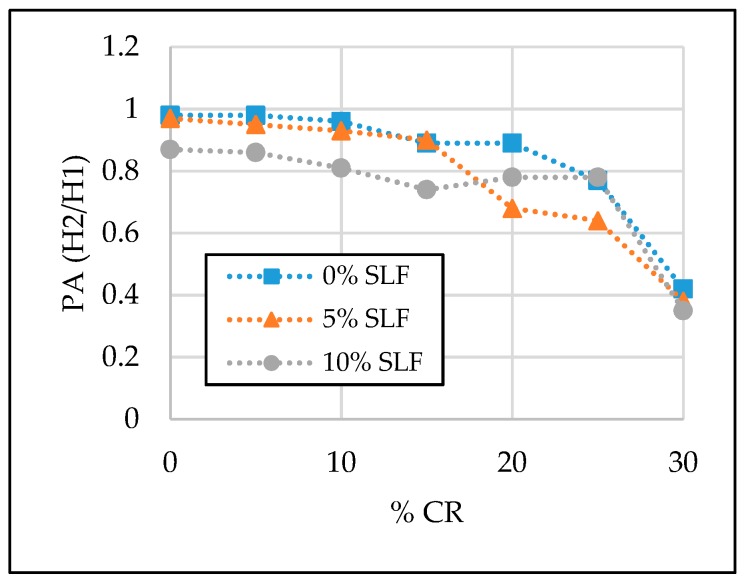
The effect of crumb rubber and silica fume on L-box values (PA = H_2_/H_1_).

The test results of the SCC properties in the fresh state are given in Table 6 and Figure 7, Figure 8, Figure 9 and Figure 10. Segregation was not observed in all tested SCC mixtures; therefore, the sieve segregation test was conducted only when required after a visual assessment of the fresh concrete paste.

#### 3.1.1. Flowability

From Table 6 and Figure 7, it can be seen that the inclusion of CR and SLF in SCC caused the reduction of its flowability. Among the 21 observed SCC mixtures, reference mixture SCC-0CR-0SLF has the highest value of the slump flow diameter, i.e., 780 mm, while minimum values of the slump flow diameter occur with mixtures SCC-30CR-5SLF and SCC-30CR-10SLF, which were 495 and 505 mm, respectively. Slump flow class (SF) was also changed according to the crumb rubber and silica fume amount in SCC. With a higher replacement level of fine aggregate, slump flow values change from class SF3 to classes SF2 and SF1, which can be considered as a negative effect of these two replacement materials on concrete flowability. Similarly, with a higher replacement level of cement with silica fume, values of the slump flow diameter take the value of flowability class SF1 and SF2. However, it can be concluded that the results of slump flow diameter are satisfied and in accordance with EFNARC guidelines, except for those of concrete mixtures SCC-30CR-5SLF and SCC-30CR-10SLF, where the values of slump flow diameter were below 550 mm, which was most likely because of the crumb rubber rough surface, surface friction, and sharp edges of the rubber particles [2,5] causing blockages.

#### 3.1.2. Viscosity

T500 (s) was used as a viscosity indicator of SCC mixtures. From Table 6 and Figure 8, it can be seen that the CR and SLF inclusion caused the enhancement in viscosity of the SCC mixtures. On the opposite hand, with the inclusion of silica fume, the T500 value was less affected. With 0% of crumb rubber, the T500 value was 1.85, 1.74 and 1.45 s with 0%, 5%, and 10% of silica fume, respectively. Both viscosity classes (VS1 and VS2) are connected with the test results of the T500 parameter; hence, the majority of the SCC mixtures with up to 15% of crumb rubber and up to 10% of silica fume can be classified as viscosity class VS1 because T500 value was below 2 s, which is exceptionally suitable for reinforced structural elements with congested reinforcement. The greatest value of T500 test results was with the 30% fine aggregate replacement and 10% cement replacement. From the given results, it can be suggested than test results with up to 20% of crumb rubber and up to 10% of silica fume are reasonable and acceptable.

#### 3.1.3. L-box and J-ring

The passing ability of fresh concrete mixture can be of great significance during the execution of concrete works at a construction site, if reinforcement is congested. Therefore, before casting concrete into molds, their passing ability was checked with two different test methods: L-box and J-ring. From test results given in Table 6 and Figure 9 and Figure 10, it can be concluded that by increasing the replacement level of natural fine aggregate and cement with crumb rubber and silica fume, the passing ability of SCC mixtures was reduced. Most of the obtained J-ring test results are between 10 and 15 mm, and the upper limit according to EFNARC guidelines for SCC is 10 mm. This can be improved with a slight increase in the superplasticizer dosage. On the other hand, the test results obtained with L-box show a satisfactory passing ability of SCC mixture with up to a 15% replacement level of natural fine aggregate with crumb rubber and with up to a 10% replacement level of cement with silica fume. Values of passing ability (PA) were higher than the limit value for the mentioned mixtures, i.e., > 0.8. However, the PA value decreased drastically when 30% of natural fine aggregate was replaced with crumb rubber. The PA was 0.42, 0.38, and 0.35 for mixtures with 30% crumb rubber and 0%, 5%, and 10% silica fume, respectively.

#### 3.1.4. Segregation Resistance

The appearance of segregation can be pronounced in high vertical-reinforced concrete elements such as columns, pylons, etc. The sieve segregation resistance test, which is defined as a percentage of concrete passed through a 5 mm sieve, can be of great help to bring conclusions regarding concrete homogeneity and concrete strength uniformity. According to the test results given in Table 6, it is clear that segregation resistance was not measured for all the concrete mixtures, since segregation was not present in all the SCC mixtures. After completion of the slump-flow, J-ring, and L-box test for each mixture, a visual inspection was made regarding whether there is water separation at the edge of the concrete or not. If there was any, the sieve segregation resistance test was made. A slight segregation was present almost in all mixtures without silica fume. Hence, viscosity-modifying admixture was added to increase segregation resistance and cohesion, in the amount of 0.24% cement mass, in order to achieve the desired segregation resistance class SR2, i.e., below 15%. According to the test results given in Table 6, it can be concluded that all SCC mixtures without silica fume have segregation resistance value (SR) within the permissible limits given by the EFNARC Guidelines. SCRC mixtures containing silica fume as a supplementary cementitious material did not have pronounced water separation at the edge of the concrete; therefore, the sieve segregation resistance test was not performed.

The test results of the SCC fresh state properties were in accordance with investigations carried out by different authors so far [8,44,60,61], where fine aggregate (0–4 mm) was replaced with crumb rubber. This behavior can be explained by the sharp edges [2,5] of rubber particles causing blockages; hence, more energy is required to move concrete paste and an enhancement in viscosity and reduction in flowability and passing ability occurs. However, with up to 15% of the fine aggregate replacement level and 5% of the cement replacement level, favorable fresh SCC properties classes can be obtained.

### 3.2. Hardened SCC Properties

**Figure 11:**
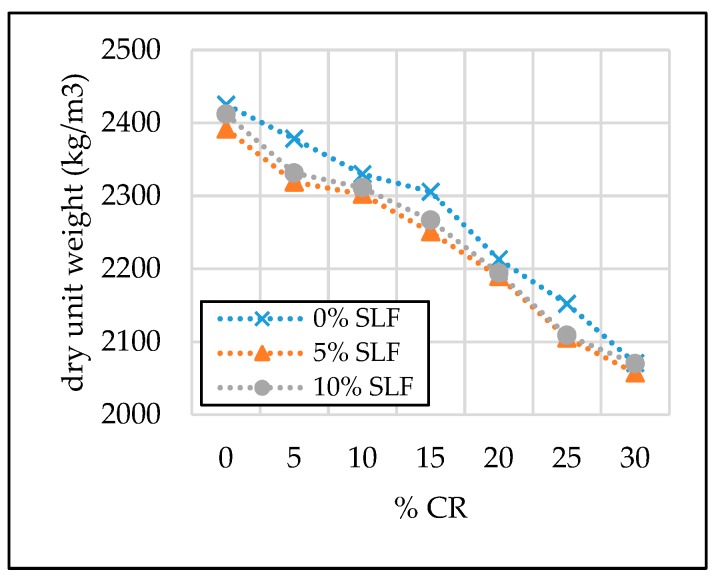
The effect of crumb rubber and silica fume on dry unit weight.

**Figure 12:**
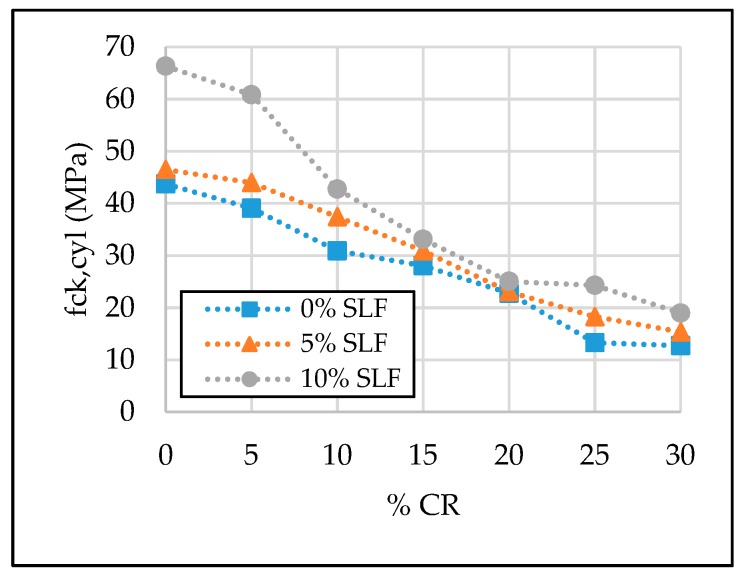
The effect of crumb rubber and silica fume on 28-day compressive strength.

**Figure 13:**
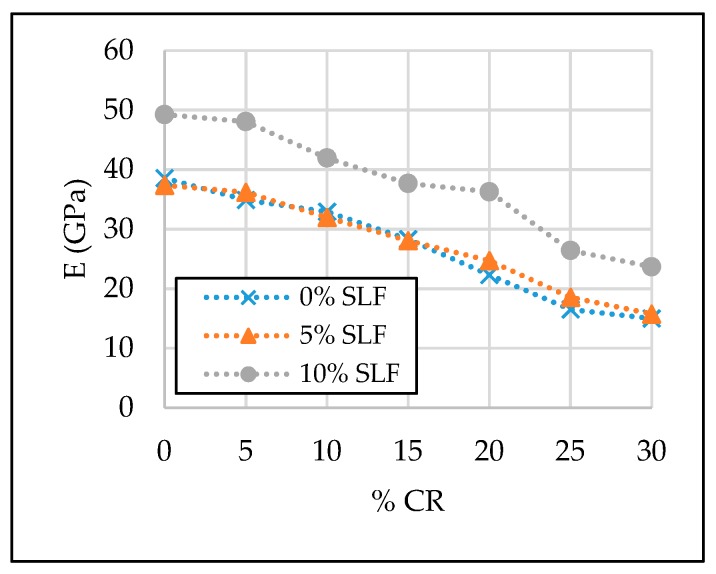
The effect of crumb rubber and silica fume on 28-day modulus of elasticity.

**Figure 14:**
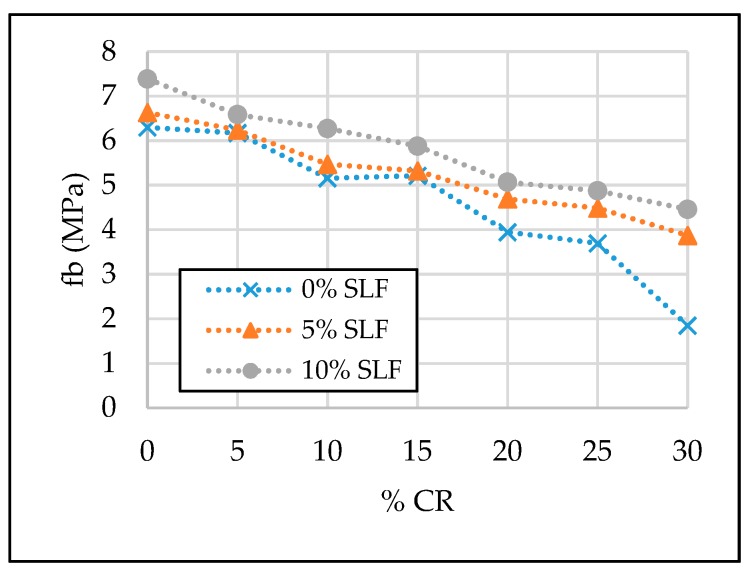
The effect of crumb rubber and silica fume on 28-day flexural strength.

The test results of the SCC properties in the hardened state are given in Table 7 and Figure 11, Figure 12, Figure 13 and Figure 14.

#### 3.2.1. Dry Unit Weight

The unit weight of SCC cylinders and prisms was measured after 28 days. The results given in Table 7 and Figure 11 show that the dry unit weight decreases with an increasing content of crumb rubber due to its low specific gravity (1.05 g/cm^3^). The relative decrease in dry unit weight was approximately 7% and 15% with 15% and 30% replacement levels of fine aggregate and 0% and 5% replacement levels of cement, respectively. An almost linear relationship between a reduction in dry unit weight and increase of replacement level of natural fine aggregate with crumb rubber was observed. Lightweight self-compacting concrete with a dry unit weight around 2069 kg/m^3^ was obtained, with 30% of the fine aggregate replacement level, which ultimately leads to a lower total mass of the concrete structures if such concrete is intended for structural purposes.

#### 3.2.2. Compressive Strength (f_ck, cyl_)

As expected, the mean value of three measurements of 28-day compressive strength was the lowest, i.e., 12.21 MPa, when the CR and SLF replacement levels were 30% and 0%, respectively. The negative impact of CR on the 28-day compressive strength can be described with a poor rubber granule–cement paste bond and with a low rubber modulus of elasticity compared to natural aggregates [5,62]. On the contrary, the mean value of three measurements of 28-day compressive strength was the greatest when the replacement level of cement with silica fume was 10%, with 0% of crumb rubber content, i.e., 66.30 MPa (Table 7, Figure 12). As a result of its pozzolanic activity, silica fume contributes to the increase in values of not only its compressive strength, but also the modulus of elasticity and flexural strength. However, according to the obtained results and due to the economic criteria, it can be suggested that silica fume should be used in combination with recycled rubber only up to a 5% replacement level of the cement. Any replacement above 5% is questionable in terms of cost-effectiveness, because of the relatively big drop in the compressive strength of SCRC cylinder specimens at higher replacement levels of fine aggregate with recycled crumb rubber. Compared to reference mixture SCC-0CR-0SLF, the value of the 28-day compressive strength of the mixture SCC-30CR-0SLF was reduced by 71%. This reduction is alleviated in case of 30% crumb rubber and 5% or 10% silica fume, but it is still significant, at 65% and 57%, respectively. Consequently, it can be seen that at a higher replacement level of fine aggregate with crumb rubber, i.e., 30%, silica fume as a supplementary cementitious material can reduce the negative effect by up to 14%. In other words, the positive effect of adding silica fume to self-compacting rubberized concrete is more noticeable on a lower fine aggregate replacement level, i.e., up to 10%. With 5% silica fume and 5% crumb rubber, the values of the 28-day compressive strength are almost equal to the compressive strength value of the reference SCC mixture. A similar occurrence is with 10% silica fume, where the improvement is even higher. In this case, the value of the 28-day compressive strength is 39% higher than the value of the reference SCC mixture. Only three values of the 28-day compressive strength were below the limit value for structural applications of 17 MPa [9], i.e., SCC-25CR-0SLF, SCC-30CR-0SLF, and SCC-30CR-5SLF. On the other hand, values of the 28-day compressive strength of the SCC mixtures labeled as SCC-15CR-5SLF and SCC-10CR-0SLF were above 30 MPa, which sound promising for the future investigation of self-compacting concrete on the structural elements and systems.

#### 3.2.3. Modulus of Elasticity (E)

Values of the 28-day modulus of elasticity were in a direct link with the compressive strength values, although in this case, the negative effect of replacing natural fine aggregate with crumb rubber is more pronounced, since with 5% of silica fume, the negative impact of rubber granules on the elasticity modulus values cannot be annulled (Table 7, Figure 13). Compared to the value of the modulus of elasticity of reference mixture SCC-0CR-0SLF, a reduction of approximately 56% can be observed with 30% of crumb rubber. The beneficial effect of replacing cement with silica fume was manifested at concrete mixtures with 0%, 5%, and 10% of crumb rubber and 10% of silica fume, where supplementary cementitious material contributed to a relative increase in the values of the modulus of elasticity by 28%, 25% and 9%, respectively. However, with 15% of crumb rubber and 0%, 5%, and 10% of silica fume, the reduction in modulus of elasticity was 27%, 27%, and 2%. An explanation for these values of modulus of elasticity of the SCC with crumb rubber and silica fume can be described in the same way as with the compressive strength values. However, the negligible positive impact of the 5% replacement level of cement with silica fume on the values of the modulus of elasticity can be reported. Nevertheless, up to a 20% fine aggregate replacement level, values of the 28-day modulus of elasticity were over 20 GPa.

#### 3.2.4. Flexural Strength (f_b_)

Flexural strength also experienced a negative impact with replacing natural fine aggregate with crumb rubber, but this was still at smaller percentage compared to the compressive strength and modulus of elasticity (Table 7, Figure 14). Thus, it can be noted that compared to the reference mixture, a maximum reduction in the value of the 28-day flexural strength was 71% for the mixture SCC-30CR-0SLF. However, this reduction is significantly less, i.e., 39% and 29%, when 5% and 10% of cement was replaced with silica fume, for an equal crumb rubber replacement level of 30%. Despite the relative reduction in the flexural strength of the SCC mixture SCC-30CR-0SLF being equal to the relative reduction in compressive strength for the same mixture, i.e., 71%, other percentages of relative reduction of the flexural strength were still much lower compared to other percentages of relative reduction of the compressive strength. For example, the relative reduction in 28-day flexural strength for SCC-15CR-5SLF was only 16%, compared to a 29% relative reduction in the 28-day compressive strength for the same mixture. Similar behavior was also reported by several different authors [7,11,32,45] who also used crumb rubber as a fine aggregate replacement.

### 3.3. Prediction Models for SCC Mechanical Properties

The experimental dataset for the present work is based on 63 values of the observed mechanical properties: three for each of 21 SCC mixtures (an experimental dataset can be provided on request). To predict the three main mechanical properties of SCC—compressive strength, modulus of elasticity and flexural strength—a multivariate regression technique was applied for each of the observed mechanical properties by using two independent variables: the amount of crumb rubber (0–30%) and silica fume (0–10%). Regression models were extracted by using software “Statistica 13” and “R”. The final models are given in Table 8.

Multivariate regression models have been developed for the partial replacement of natural fine aggregate with crumb rubber (0–30%) and the partial replacement of cement with silica fume (0–10%). For the estimation of unknown parameters of modulus of elasticity, the least squares (LS) method was used. However, when applying the LS method for the estimation of unknown parameters of the other two mechanical properties, i.e., compressive strength and flexural strength, the variability of the variance of the residuals was observed, depending on the silica fume replacement level. Hence, for an estimation of unknown parameters of compressive strength and flexural strength, the weighted least squares (WLS) method was used. In these cases, weights for WLS were determined by modeling squares of residuals in the initial model with the silica fume replacement level. The results of the regression analysis and estimated coefficients for all three mechanical properties are given in Table 9, Table 10 and Table 11.

**Figure 15:**
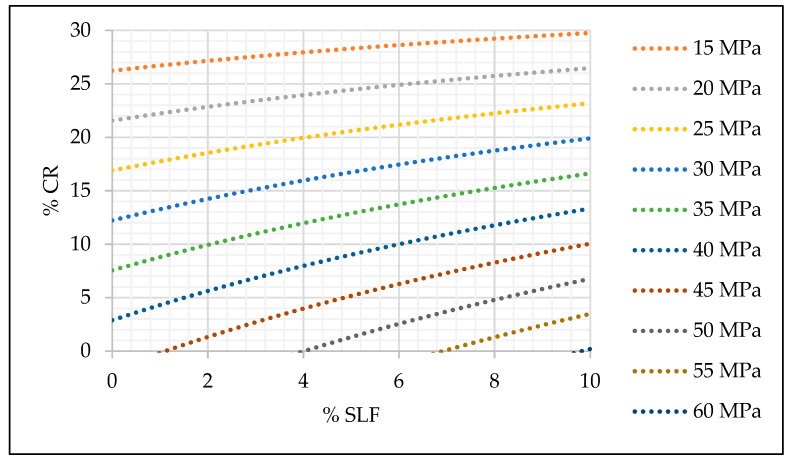
Prediction model for SCC compressive strength (fck = 43.09240 − 1,07104 × cr + 1.72105 × slf − 0.04515 × cr × slf).

**Figure 16:**
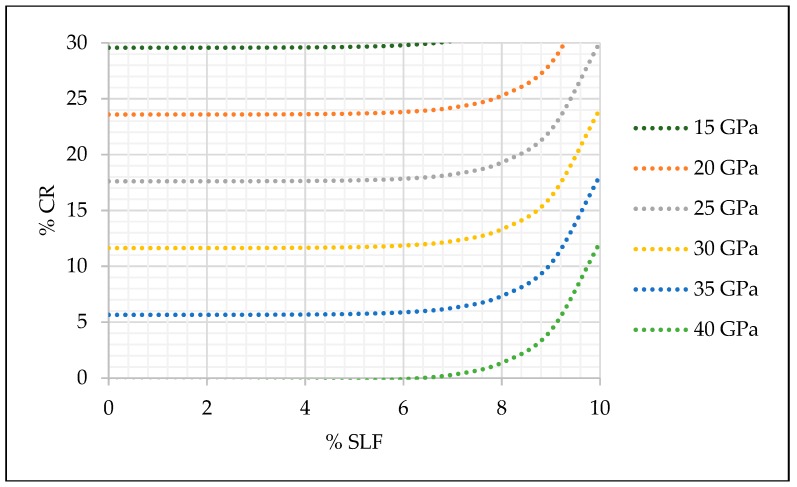
Prediction model for SCC modulus of elasticity (E = 39.7295 − 0.836401 × cr + 0.000473885 × exp(slf).

**Figure 17:**
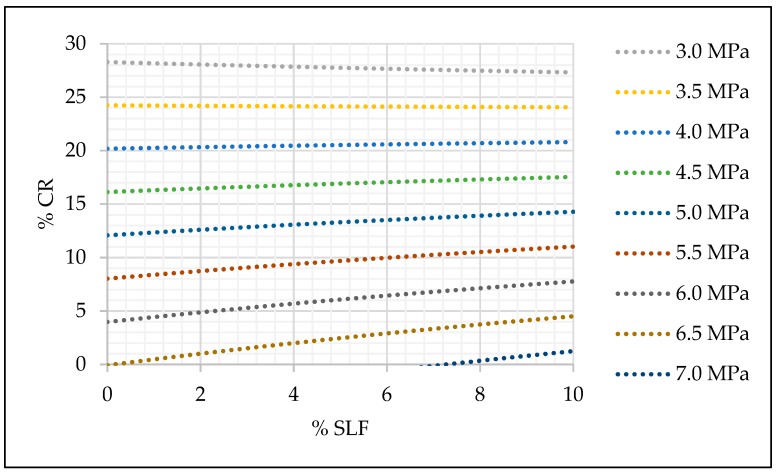
Prediction model for SCC flexural strength (fb = 6.489658 − 0.123341 × cr + 0.070092 × slf − 0.003003 × cr × slf).

With estimated coefficients of the selected models, the relation between mechanical properties and the amount of crumb rubber and silica fume were presented graphically in Figure 15, Figure 16 and Figure 17.

For the chosen regression models, non-constant variance score tests (NCV test) were conducted [63], and they do not show the presence of heteroscedasticity. Furthermore, the Shapiro–Wilk test [64] did not suggest a deviation of standardized residuals from normality (p-values were 0.6533, 0.4971, and 0.1354 for regression models of compressive strength, modulus of elasticity, and flexural strength, respectively).

The practical application of prediction models and the graph given in Figure 15, Figure 16 and Figure 17 can be simple, and it is explained as follows. According to the designed and required concrete class, engineers can easily select the replacement level of fine aggregate and cement and still maintain the desired concrete strength. For example, if a concrete compressive strength of 30 MPa is required, 10% of fine aggregate replacement and 0% of cement replacement can be made. For the same concrete class, different percentage of replacement can be made, i.e., 15% of fine aggregate and 5% of silica fume.

The positive impact of silica fume on SCRC compressive strength, even with a minor replacement level of cement, can be seen from Figure 15. However, Figure 16 and Figure 17 point to a slightly different conclusion where the impact of a minor replacement level of silica fume on the modulus of elasticity and flexural strength is questionable, and these replacement levels still need to be additionally investigated in order to investigate the influence of the smaller replacement levels of silica fume on these two mechanical properties of SCRC.

### 3.4. Model Verification by Comparison with Other Experimental Studies

In this section, 12 experimental studies from the literature are compared with the developed models: 7 for the compressive strength model [5,6,11,42,43,65,66], 6 for the modulus of elasticity model [6,11,12,43,44,65], and 8 for the flexural strength model [5,6,11,31,42,44,65,67]. To avoid any unnecessary disagreements and inconsistencies with the proposed model, the results of experimental studies of other researchers were carefully selected as follows:

- Concrete type is SCC, not traditional concrete
- CR is replacing FA, not CA
- CR is replacing fine aggregate by total aggregate volume/fine aggregate volume, not by total aggregate weight/fine aggregate weight
- SLF is replacing cement by total cement mass, not by total powder/binder content
- Avoid experimental investigation with multiple supplementary cementitious material, such as combination of SLF and metakaolin, fly ash, or ground granulated blast furnace slag
- Observed CR and SLF replacement level is 0–30% and 0–10%, respectively
- SCC compressive strength and modulus of elasticity is calculated from the experimental investigation of the cylinder specimen, whilst SCC flexural strength is calculated from the prism specimens, respectively.

**Figure 18:**
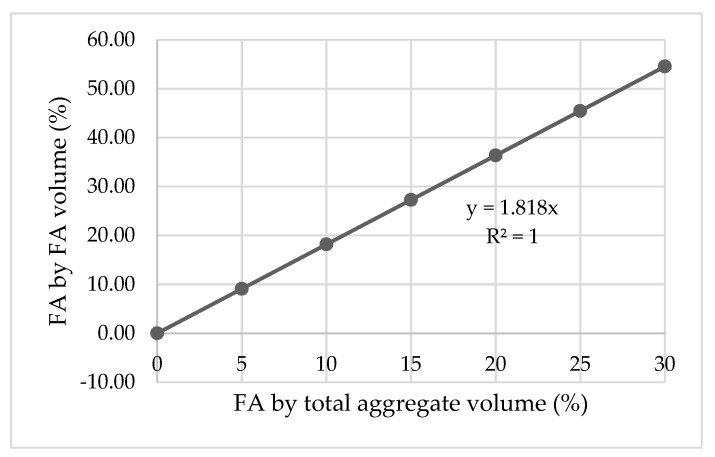
Conversion coefficient (c = 1/1.818 = 0.55).

As it was mentioned in Section 2, cement was replaced with SLF by total cement mass, while FA was replaced with CR by total aggregate volume. However, in all the experimental studies of the other researchers used for comparison, FA was replaced with CR by FA volume. Hence, to adjust the percentages of the replacement levels, a conversion coefficient needs to be applied on the CR percentage level in all cases where CR was used. This conversion coefficient can be easily obtained through a linear relationship of the FA by total aggregate volume (%) and FA by FA volume (%), which was used in model creation (Figure 18).

**Figure 19:**
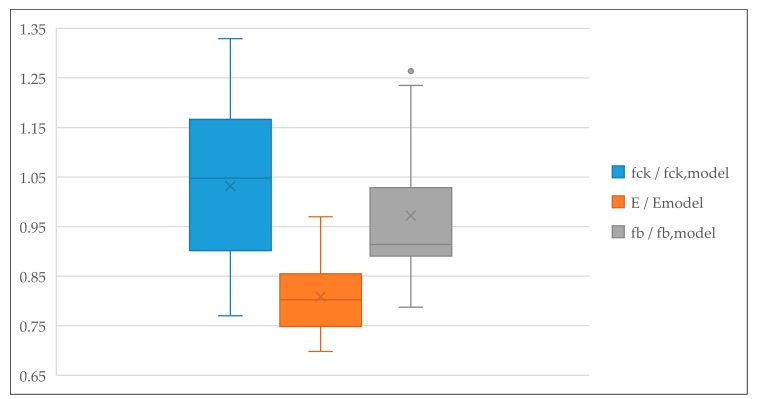
Verification of the prediction model—graphical interpretation.

Verification of the prediction models based on existing test results from the available literature is given in Table 12, Table 13 and Table 14 and Figure 19. The verifications of the developed models for the compressive strength and flexural strength yield good agreement with the experimental results of the other research studies. Ratio f_ck_/f_ck, model_ obtained from 39 verification values has a range of values from 0.77 to 1.33, with a total average of 1.03 and median of 1.05. Ratio f_b_/f_b, model_ obtained from 44 values has a range from 0.79 to 1.27, with a total average of 0.97 and median of 0.91. However, the verification of the developed model for the modulus of elasticity is a little less reliable than the other models. Ratio E/E_model_ obtained from 30 values has a range from 0.7 to 0.97, with a total average of 0.81 and median of 0.80.

In all three cases, factors that are possibly affecting the difference between experiment values and model values are listed as follows:

- Material properties (cement, aggregate, fillers, SCMs, recycled rubber, chemical admixtures, etc.)
- Mix proportions (w/b ratio, w/p ratio, binder content, SCMs content, FA to CA ratio, etc.)
- Mixing procedure and curing conditions (specimen preparation)
- Test methods (standards and specimen dimensions)

For future investigations and further development of the SCC model with SLF and CR, it can be suggested that additional laboratory work needs to be done, with a variation of more than two variables, such as w/b ratio, binder content, cement type, etc. With additional variables included in the models, a larger database of the experimental results of the other researches can be used in the model verification process.

## 4. Discussion

In the present study, the fresh and hardened properties of SCC mixtures with different amounts of crumb rubber (0–30%) and silica fume (0–10%) were investigated. Furthermore, the test results of SCC in the hardened state were used for creation of the prediction models. Developed models were verified with 12 experimental studies. Several conclusions arise from the conducted experimental research and development of the statistical models:

- An increase in the replacement level of fine aggregate with crumb rubber and cement with silica fume had a negative impact on the flowability of SCC mixtures; however, slump flow values were within acceptable limits for SCC mixtures with up to 25% of crumb rubber and 10% of silica fume.
- SCC mixtures with up to 15% of crumb rubber and up to 10% of silica fume can be classified as viscosity class VS1 (T500 < 2 s).
- J-ring and L-box test results show a satisfactory passing ability of SCC mixture with up to 15% of crumb rubber and 10% of silica fume.
- Segregation was not present when 5% and 10% of cement was replaced with silica fume, but slight segregation was present in all mixtures without silica fume.
- A linear relationship between reduction in the dry unit weight and increase of replacement levels of natural fine aggregate with crumb rubber was observed.
- Considering the compressive strength of SCC, at a 30% replacement level of fine aggregate with crumb rubber, silica fume can reduce the negative effect by up to 14%.
- Values of the 28-day compressive strength of the SCC mixtures SCC-15CR-5SLF and SCC-10CR-0SLF were above 30 MPa, which can be taken into account if there is an intention for future investigation of self-compacting concrete on the structural elements and systems. Still, according to the obtained results, it can be suggested that the silica fume should be used in combination with recycled rubber only up to a 5% replacement level of the cement.
- Values of the 28-day modulus of elasticity were over 20 GPa with a fine aggregate replacement level of up to 20%.
- Replacing natural fine aggregate with crumb rubber also caused negative effects on the value of the flexural strength, but still at a smaller percentage compared to the compressive strength and modulus of elasticity.
- The positive impact of replacing cement with silica fume is more obvious for a lower replacement level of the natural fine aggregate with crumb rubber, i.e., up to 10%.
- According to the developed model for the prediction of the compressive strength, with 10% and 15% of CR and 0% and 5% of SLF, a concrete compressive strength of 30 MPa can be obtained.
- The verifications of the prediction models for the compressive strength and flexural strength give good agreement with the experimental results of the other research studies, while the prediction model for the modulus of elasticity is less reliable than that of the other two models.
- Values of the experiment/model ratio are affected by several factors such as the material properties, mix proportions, mixing procedure, and test methods.

## 5. Conclusions

The following are the overall conclusions drawn out of the obtained test results and observations made in this study:

- Favorable fresh and hardened SCC properties can be obtained with up to 15% of crumb rubber and 5% of silica fume.
- Models for the prediction of mechanical properties of SCC with crumb rubber and silica fume were developed and successfully verified.
- Future research directions:
- With an extensive laboratory work, create a database for defining an extended model with more than two variables included, such as the w/b ratio, binder content, cement type etc.
- Detailed analysis of the durability, thermal, long-term and micro-level properties (SEM) needs to be done, and thereafter full-scale models of SCRC beams, columns, and frames with selected optimal SCC mixtures need to be investigated.

## Figures and Tables

**Table 1:** Chemical properties of cement.

Chemical Compound	% of Mass	Chemical Compound	% of Mass
CaO	61.24	MgO	2.84
SiO_2_	21.33	MnO	0.14
Al_2_O_3_	5.69	K_2_O	0.74
Fe_2_O_3_	2.27	Na_2_O	0.37
SO_3_	3.52	LOI	1.52

**Table 2:** Physical and chemical properties of silica fume.

Color and Form	Main Active Component	Density (g/cm^3^)	SiO_2_ (%)	Specific Surface (m^2^/g)	Primary Graining (μm)	Maximum Consumption (% of cement weight)
Gray powder	Amorphous SiO_2_	2.2	96 ± 1.5	20.5	0.1–0.3	11

**Table 3:** Properties of superplasticizer (SP) and viscosity-modifying admixture (VMA).

Property	SP–Energy FM500	VMA–Premadd Stabilisator X
Shape	liquid	liquid
Color	amber	white, milky
Density (kg/L)	1.08 ± 0.02	1.04 ± 0.02
pH Value	3.5 ± 2.0	7.0 ± 2.0

**Table 4:** Mix proportions for self-compacting concrete (kg/m^3^). CA: coarse aggregate, CR: crumb rubber, FA: fine aggregate, SLF: silica fume, w/b: water to binder.

No	Mixture ID	Cement	SLF	Filler	FA	CA	CR	w/b	SP (%)	VMA (%)
1	SCC-0CR-0SLF	450	0	80	941.61	817.65	0	0.4	1.25	0.24
2	SCC-5CR-0SLF	450	0	80	853.6	817.65	33.12	0.4	1.25	0.24
3	SCC-10CR-0SLF	450	0	80	765.59	817.65	66.24	0.4	1.25	0.24
4	SCC-15CR-0SLF	450	0	80	677.58	817.65	99.37	0.4	1.25	0.24
5	SCC-20CR-0SLF	450	0	80	589.57	817.65	132.49	0.4	1.25	0.24
6	SCC-25CR-0SLF	450	0	80	501.57	817.65	165.61	0.4	1.25	0.24
7	SCC-30CR-0SLF	450	0	80	413.55	817.65	198.73	0.4	1.25	0.24
8	SCC-0CR-5SLF	427.50	22.5	80	937.27	813.87	0	0.4	1.25	—
9	SCC-5CR-5SLF	427.50	22.5	80	849.66	813.87	32.97	0.4	1.25	—
10	SCC-10CR-5SLF	427.50	22.5	80	762.06	813.87	65.94	0.4	1.25	—
11	SCC-15CR-5SLF	427.50	22.5	80	674.46	813.87	98.91	0.4	1.25	—
12	SCC-20CR-5SLF	427.50	22.5	80	586.85	813.87	131.88	0.4	1.25	—
13	SCC-25CR-5SLF	427.50	22.5	80	499.25	813.87	164.85	0.4	1.25	—
14	SCC-30CR-5SLF	427.50	22.5	80	411.64	813.87	197.81	0.4	1.25	—
15	SCC-0CR-10SLF	405	45	80	932.92	810.05	0	0.4	1.25	—
16	SCC-5CR-10SLF	405	45	80	845.72	810.05	32.82	0.4	1.25	—
17	SCC-10CR-10SLF	405	45	80	758.53	810.05	65.63	0.4	1.25	—
18	SCC-15CR-10SLF	405	45	80	671.33	810.05	98.45	0.4	1.25	—
19	SCC-20CR-10SLF	405	45	80	584.13	810.05	131.26	0.4	1.25	—
20	SCC-25CR-10SLF	405	45	80	496.93	810.05	164.08	0.4	1.25	—
21	SCC-30CR-10SLF	405	45	80	409.79	810.05	196.90	0.4	1.25	—

**Table 5:** Tested self-compacting concrete (SCC) fresh and hardened state properties.

State	SCC Property	Test and Specimen Type
Fresh state	Flowability (d)	Slump flow test
	Viscosity (T500)	
	Passing ability (PL and PJ)	L-box test
		J-ring test
	Segregation (SR)	Sieve segregation test
Hardened state	Compressive strength (f_ck,cyl_)	Cylinder specimens dim. Ø150 × 300 mm
	Modulus of elasticity (E)	
	Flexural strength (f_b_)	Prism specimens dim. 100 × 100 × 400 mm

**Table 6:** Test results of fresh SCC properties.

SCC Fresh State Property				Viscosity			Flowability			Passing Ability					Segregation Resistance		
No	Mixture	CR	SLF	T500			Slump Flow			J-ring		L-box			Sieve Segregation		
		(%)	(%)	(s)	Class		d (mm)	Class		PJ (mm)	Class	PA (H_2_/H_1_)	Class		SR (%)	Class	
1	SCC-0CR-0SLF	0	0	1.9	<2	VS1	780	760–850	SF3	10	10	0.98	>0.80	PA2	11.83	<15	SR2
2	SCC-5CR-0SLF	5	0	1.9	<2	VS1	760	760–850	SF3	12.5	>10	0.98	>0.80	PA2	10.1	<15	SR2
3	SCC-10CR-0SLF	10	0	1.9	<2	VS1	750	660–750	SF2	13	>10	0.96	>0.80	PA2	8.75	<15	SR2
4	SCC-15CR-0SLF	15	0	2.3	>2	VS2	685	660–750	SF2	15	>10	0.89	>0.80	PA2	8.33	<15	SR2
5	SCC-20CR-0SLF	20	0	2.7	>2	VS2	690	660–750	SF2	25	>10	0.89	>0.80	PA2	3.83	<15	SR2
6	SCC-25CR-0SLF	25	0	3	>2	VS2	655	550–650	SF1	26	>10	0.77	<0.80	PA2	3.43	<15	SR2
7	SCC-30CR-0SLF	30	0	6.5	>2	VS2	555	550–650	SF1	41.25	>10	0.42	<0.80	PA2	2.58	<15	SR2
8	SCC-0CR-5SLF	0	5	1.7	<2	VS1	750	660–750	SF2	10	0	0.97	>0.80	PA2	—	<15	SR2
9	SCC-5CR-5SLF	5	5	1.9	<2	VS1	690	660–750	SF2	16.25	>10	0.95	>0.80	PA2	—	<15	SR2
10	SCC-10CR-5SLF	10	5	1.9	<2	VS1	682	660–750	SF2	17.25	>10	0.93	>0.80	PA2	—	<15	SR2
11	SCC-15CR-5SLF	15	5	1.9	<2	VS1	644	550–650	SF1	20	>10	0.9	>0.80	PA2	—	<15	SR2
12	SCC-20CR-5SLF	20	5	2.5	>2	VS2	600	550–650	SF1	28.25	>10	0.68	<0.80	PA2	—	<15	SR2
13	SCC-25CR-5SLF	25	5	3.2	>2	VS2	555	550–650	SF1	29.75	>10	0.64	<0.80	PA2	—	<15	SR2
14	SCC-30CR-5SLF	30	5	8.6	>2	VS2	495	—	—	34	>10	0.38	<0.80	PA2	—	<15	SR2
15	SCC-0CR-10SLF	0	10	1.5	<2	VS1	690	660–750	SF2	6.25	<10	0.87	>0.80	PA2	—	<15	SR2
16	SCC-5CR-10SLF	5	10	1.6	<2	VS1	685	660–750	SF2	9.5	<10	0.86	>0.80	PA2	—	<15	SR2
17	SCC-10CR-10SLF	10	10	1.7	<2	VS1	670	660–750	SF2	9.75	<10	0.81	>0.80	PA2	—	<15	SR2
18	SCC-15CR-10SLF	15	10	1.8	<2	VS1	670	660–750	SF2	11	>10	0.74	<0.80	PA2	—	<15	SR2
19	SCC-20CR-10SLF	20	10	1.9	<2	VS1	685	660–750	SF2	12	>10	0.78	<0.80	PA2	—	<15	SR2
20	SCC-25CR-10SLF	25	10	2.1	>2	VS2	655	550–650	SF1	13.75	>10	0.78	<0.80	PA2	—	<15	SR2
21	SCC-30CR-10SLF	30	10	4.4	>2	VS2	505	—	—	30.5	>10	0.35	>0.80	PA2	—	<15	SR2

**Table 7:** Test results of hardened SCC properties.

R.b.	Mixture	Dry Unit Weight (kg/m^3^)	f_ck,cyl_ (MPa)			E (GPa)			f_b_ (MPa)		
		Mean	Mean	st dev.	CV %	Mean	st dev.	CV %	Mean	st dev.	CV %
1	SCC-0CR-0SLF	2424.89	43.72	2.54	5.82%	38.58	2.95	5.82%	6.30	0.14	2.23%
2	SCC-5CR-0SLF	2378.23	39.04	3.10	7.93%	34.89	2.27	6.50%	6.17	0.22	3.55%
3	SCC-10CR-0SLF	2329.60	30.88	1.82	5.89%	32.92	4.70	14.26%	5.15	0.10	2.01%
4	SCC-15CR-0SLF	2305.52	28.04	1.41	5.04%	28.30	1.28	4.52%	5.21	0.18	3.37%
5	SCC-20CR-0SLF	2212.76	22.76	1.29	5.67%	22.30	2.04	9.15%	3.95	0.08	1.96%
6	SCC-25CR-0SLF	2151.96	13.28	1.98	14.93%	16.50	2.03	12.30%	3.69	0.43	11.58%
7	SCC-30CR-0SLF	2070.74	12.71	0.52	4.05%	14.99	1.30	8.66%	1.85	0.27	14.56%
8	SCC-0CR-5SLF	2391.46	46.48	5.05	10.87%	37.37	4.03	10.79%	6.63	0.25	3.79%
9	SCC-5CR-5SLF	2318.86	43.98	3.11	7.07%	36.20	3.25	8.97%	6.24	0.02	0.32%
10	SCC-10CR-5SLF	2302.14	37.42	1.30	3.48%	31.97	0.60	1.88%	5.47	0.16	2.85%
11	SCC-15CR-5SLF	2250.54	30.89	2.08	6.72%	28.06	3.37	12.01%	5.32	0.24	4.53%
12	SCC-20CR-5SLF	2189.49	23.08	1.76	7.65%	24.71	0.06	0.24%	4.69	0.07	1.44%
13	SCC-25CR-5SLF	2105.02	18.26	0.10	0.55%	18.54	4.15	22.38%	4.48	0.09	1.99%
14	SCC-30CR-5SLF	2057.35	15.35	0.95	6.20%	15.75	1.43	6.20%	3.87	0.06	1.44%
15	SCC-0CR-10SLF	2411.98	66.30	1.39	2.09%	49.26	1.10	2.23%	7.38	0.23	3.07%
16	SCC-5CR-10SLF	2331.30	60.83	5.23	8.60%	48.06	4.18	8.69%	6.58	0.28	4.31%
17	SCC-10CR-10SLF	2311.11	42.73	4.48	10.48%	41.94	2.70	6.44%	6.27	0.17	2.75%
18	SCC-15CR-10SLF	2266.57	33.11	3.22	9.72%	37.69	2.78	7.39%	5.88	0.26	4.35%
19	SCC-20CR-10SLF	2194.11	25.04	1.85	7.38%	36.28	4.62	12.75%	5.07	0.03	0.58%
20	SCC-25CR-10SLF	2108.65	24.28	2.47	10.16%	26.42	3.40	12.87%	4.87	0.19	4.00%
21	SCC-30CR-10SLF	2069.84	18.96	4.29	22.64%	23.68	1.10	4.64%	4.46	0.19	4.35%

**Table 8:** Final regression models for the prediction of the mechanical properties with corresponding estimated regression equations.

Mechanical Property	Final Regression Model
Compressive strength (f_ck_)	b0 + b1 × cr + b2 × slf + b3 × cr × slf + ε
Flexural strength (f_b_)	b0 + b1 × cr + b2 × slf + b3 × cr × slf + ε
Modulus of elasticity (E)	b0 + b1 × cr + b2 × exp(slf) + ε

b0, b1, b2, b3—regression coefficients. ε—model error. cr—replacement level of natural fine aggregate with crumb rubber (%). slf—replacement level of cement with silica fume (%).

**Table 9:** Results of the regression analysis and coefficient estimation—compressive strength (fck = 43.09240 − 1.07104 × cr + 1.72105 × slf − 0.04515 × cr × slf).

Coefficient	Estimate	Std. Error	Pr (>|t|)	2.5%	97.5%	Adj. R^2^
b0	43.09240	0.81541	<2e^−16^	41.46076	44.72403	0.9391
b1	−1.07104	0.04523	<2e^−16^	−1.16154	−0.98053	
b2	1.72105	0.21431	4.86e^−11^	1.29221	2.14988	
b3	−0.04515	0.01189	0.00034	−0.06893	−0.02136	

**Table 10:** Results of the regression analysis and coefficient estimation—modulus of elasticity (E = 39.7295 − 0.836401 × cr + 0.000473885 × exp(slf).

Coefficient	Estimate	Std. Error	Pr (>|t|)	2.5%	97.5%	Adj. R^2^
b0	3.973e^+1^	6.934e^−1^	<2e^−16^	38.34327	41.11725	0.922
b1	−8.364e^−1^	3.576e^−2^	<2e^−16^	−0.90794	−0.76489	
b2	4.739e^−4^	3.455e^−5^	<2e^−16^	0.00040	0.00054	

**Table 11:** Results of the regression analysis and coefficient estimation – flexural strength (fb = 6.489658 − 0.123341 × cr + 0.070092 × slf − 0.003003 × cr × slf).

Coefficient	Estimate	Std. Error	Pr (>|t|)	2.5%	97.5%	Adj. R^2^
b0	6.48965	0.16031	<2e^−16^	6.16887	6.81044	0.9372
b1	−0.12334	0.00889	<2e^−16^	−0.14113	−0.10554	
b2	0.07009	0.01860	0.00038	0.03286	0.10732	
b3	0.00300	0.00103	0.00509	0.00093	0.00506	

**Table 12:** Verification of the prediction model for the compressive strength (f_ck_).

Reference	CR (%)	SLF (%)	CR after Conversion (%)	f_ck_ (MPa)	f_ck,model_ (MPa)	f_ck_/f_ck,model_
[6]	0	0	0	52.95	43.09	1.23
	5	0	2.75	44.54	40.15	1.11
	10	0	5.50	42.09	37.20	1.13
	15	0	8.25	37.35	34.26	1.09
	20	0	11.00	30.69	31.31	0.98
	25	0	13.75	28.83	28.36	1.02
	30	0	16.50	24.73	25.42	0.97
	20	0	11.00	32.81	31.31	1.05
	30	0	16.50	27.05	25.42	1.06
[5]	0	10	0	80.15	60.30	1.33
	5	10	2.75	62.12	56.12	1.11
	10	10	5.50	49.11	51.93	0.95
	15	10	8.25	41.5	47.74	0.87
	20	10	11.00	33.54	43.55	0.77
	5	10	2.75	58.1	56.12	1.04
	20	10	11.00	35.12	43.55	0.81
	25	10	13.75	30.76	39.37	0.78
[11]	0	0	0	53.5	43.09	1.24
	5	0	2.75	47.07	40.15	1.17
	10	0	5.50	43.39	37.20	1.17
	15	0	8.25	38.45	34.26	1.12
	20	0	11.00	32.81	31.31	1.05
	25	0	13.75	27.05	28.36	0.95
	30	0	16.50	21.1	25.42	0.83
[65]	0	0	0	50.8	43.09	1.18
	0	5	0	61.2	51.70	1.18
	0	10	0	62.1	60.30	1.03
[42]	0	0	0	38.85	43.09	0.90
	0	2	0	40.48	46.53	0.87
	0	4	0	45.41	49.98	0.91
	0	6	0	47.94	53.42	0.90
	0	8	0	50.03	56.86	0.88
	0	10	0	52.32	60.30	0.87
[66]	0	0	0	51.8	43.09	1.20
	0	10	0	56.5	60.30	0.94
	0	0	0	52.5	43.09	1.22
	0	10	0	63.4	60.30	1.05
[43]	0	0	0	50.8	43.09	1.18
	0	10	0	67.2	60.30	1.11

**Table 13:** Verification of the prediction model for modulus of elasticity (E).

Reference	CR (%)	SLF (%)	CR after Conversion (%)	E (GPa)	E_model_ (GPa)	E/E_model_
[6]	0	0	0	33.61	39.73	0.85
	5	0	2.75	31.51	37.43	0.84
	10	0	5.50	30.78	35.13	0.88
	15	0	8.25	27.56	32.83	0.84
	20	0	11.00	23.14	30.53	0.76
	25	0	13.75	23.01	28.23	0.82
	30	0	16.50	20	25.93	0.77
	20	0	11.00	24.1	30.53	0.79
	30	0	16.50	22.03	25.93	0.85
[11]	0	0	0	33.59	39.73	0.85
	5	0	2.75	32.56	37.43	0.87
	10	0	5.50	30.86	35.13	0.88
	15	0	8.25	27.23	32.83	0.83
	20	0	11.00	24.1	30.53	0.79
	25	0	13.75	22.03	28.23	0.78
	30	0	16.50	18.1	25.93	0.70
[44]	0	0	0	29.37	39.73	0.74
	5	0	2.75	27.54	37.43	0.74
	10	0	5.50	25.71	35.13	0.73
	15	0	8.25	24.66	32.83	0.75
	20	0	11.00	21.97	30.53	0.72
	25	0	13.75	20.02	28.23	0.71
	30	0	16.50	18.7	25.93	0.72
[65]	0	0	0	36.2	39.73	0.91
	0	5	0	38.6	39.80	0.97
	0	10	0	38.9	50.17	0.78
[12]	0	0	0	33	39.73	0.83
	22.2	0	12.21	26.6	29.52	0.90
[43]	0	0	0	36.2	39.73	0.91
	0	10	0	38.6	50.17	0.77

**Table 14:** Verification of the prediction model for modulus of elasticity (f_b_).

Reference	CR (%)	SLF (%)	CR after Conversion (%)	f_b_ (MPa)	f_b,model_ (MPa)	f_b_/f_b,model_
[31]	0	0	0	5.65	6.49	0.87
	15	0	8.25	6.48	5.47	1.18
	20	0	11.00	6.2	5.13	1.21
[6]	0	0	0	5.78	6.49	0.89
	5	0	2.75	5.58	6.15	0.91
	10	0	5.50	5.28	5.81	0.91
	15	0	8.25	5.01	5.47	0.92
	20	0	11.00	4.65	5.13	0.91
	25	0	13.75	4.37	4.79	0.91
	30	0	16.50	3.96	4.45	0.89
	20	0	11.00	5	5.13	0.97
	30	0	16.50	4.25	4.45	0.95
[5]	5	10	2.75	8.36	6.77	1.24
	10	10	5.50	6.83	6.35	1.08
	15	10	8.25	6.1	5.93	1.03
	20	10	11.00	5.65	5.50	1.03
	5	10	2.75	7.3	6.77	1.08
	20	10	11.00	5.92	5.50	1.08
	25	10	13.75	5.36	5.08	1.05
[11]	0	0	0	5.92	6.49	0.91
	5	0	2.75	5.6	6.15	0.91
	10	0	5.50	5.52	5.81	0.95
	15	0	8.25	5.19	5.47	0.95
	20	0	11.00	5	5.13	0.97
	25	0	13.75	4.25	4.79	0.89
	30	0	16.50	3.85	4.45	0.86
[44]	0	0	0	5.74	6.49	0.88
	5	0	2.75	5.48	6.15	0.89
	10	0	5.50	5.12	5.81	0.88
	15	0	8.25	4.82	5.47	0.88
	20	0	11.00	4.57	5.13	0.89
	25	0	13.75	4.27	4.79	0.89
	30	0	16.50	3.92	4.45	0.88
[65]	0	0	0	8.2	6.49	1.26
	0	5	0	8.7	6.84	1.27
	0	10	0	8.8	7.19	1.22
[67]	0	0	0	6.59	6.49	1.02
	0	5	0	6.59	6.84	0.96
[43]	0	0	0	5.11	6.49	0.79
	0	2	0	5.61	6.63	0.85
	0	4	0	6.06	6.77	0.90
	0	6	0	6.22	6.91	0.90
	0	8	0	6.55	7.05	0.93
	0	10	0	6.74	7.19	0.94
